# Chitosan-coated mesoporous silica nanoparticles for pH-responsive doxorubicin delivery in head and neck cancer

**DOI:** 10.3389/fbioe.2026.1905837

**Published:** 2026-09-15

**Authors:** Andrej Jenča, Elham Saberian, Janka Jenčová, Adriána Petrášová, Andrej Jenča, Hadi Zare-Zardini, Olexandr Kurmyshov, Naďa Homzová, Kornélia Pandyová, Mária Futejová, Azadeh Emami, Mohsen Nabi Meybodi

**Affiliations:** 1 Clinic of Stomatology and Maxillofacial Surgery, Academy of Košice, Pavol Jozef Šafárik University, Košice, Slovakia; 2 Department of Biomedical Engineering, Meybod University, Meybod, Iran; 3 Traditional Pharmacy and Pharmaceutical Sciences Research Center, and Department of pharmaceutics, Faculty of Pharmacy, Shahid Sadoughi University of Medical Sciences, Yazd, Iran.

**Keywords:** chitosan, doxorubicin, head and neck squamous cell carcinoma, pH-sensitive, silica nanoparticles

## Abstract

**Introduction:**

Head and neck squamous cell carcinoma (HNSCC) is among the most common cancers worldwide. Despite therapeutic advances, patient survival remains low due to recurrence, metastasis, and the severe side effects of conventional chemotherapy. Doxorubicin (DOX) is an effective drug; however, its cardiotoxicity and non-specific distribution limit its clinical use. This study aimed to design a pH-sensitive smart nanocarrier based on chitosan-coated mesoporous silica nanoparticles (MSN-DOX@CS) for targeted DOX release in the acidic tumor environment.

**Methods:**

The nanoparticles were synthesized by the sol–gel method and coated with chitosan. Physicochemical characteristics (FESEM, DLS, FTIR), drug release at pH 7.4 and 5.5, and cytotoxicity (MTT) on HNSCCs were evaluated.

**Results:**

The results showed that the nanoparticles are semi-spherical (∼145 nm) with a positive surface charge (+28.6 mV). Drug release at blood pH was only 18%, while at tumor pH it exceeded 76%. The empty nanocarrier was non-toxic (>90% cell survival), and the drug-loaded version exhibited greater toxicity than the free drug at 48 h (*p* < 0.05).

**Discussion:**

In conclusion, MSN-DOX@CS is presented as a safe and effective platform for targeted chemotherapy of HNSCC, with reduced systemic side effects and increased intracellular efficacy.

## 1 Introduction

Head and neck squamous cell carcinoma (HNSCC) is among the most common malignancies worldwide, affecting hundreds of thousands of people each year (Barsouk et al., 2023). Despite significant advances in conventional treatments such as surgery, radiotherapy, and systemic chemotherapy, the survival rate for patients with HNSCC remains low due to late diagnosis, local recurrence, and metastasis (Mordzińska-Rak et al., 2025; Ganesan et al., 2024). In addition, conventional chemotherapies have serious limitations because they lack selectivity between cancerous and healthy cells, and are associated with severe side effects that significantly reduce patients’ quality of life (Liu et al., 2024; Jenča et al., 2026).

Doxorubicin (DOX) is one of the most effective and widely used anthracycline chemotherapeutic drugs for treating a broad range of cancers (Xia and King, 2025). However, the clinical use of this potent drug is severely limited by its non-specific distribution in the body, short half-life in circulation, and dose-dependent side effects, particularly irreversible cardiotoxicity (Tacar et al., 2013; Sinha et al., 2025). Therefore, the development of novel, targeted drug delivery systems that can deliver the drug directly to tumor tissue and minimize systemic side effects is an urgent clinical need (Bhadran et al., 2025). Furthermore, beyond its clinical significance, DOX possesses ideal physicochemical characteristics for evaluating the performance of mesoporous silica systems. Its molecular size is compatible with the pore diameter of MSNs, allowing high loading capacity, while its primary amine groups contribute to pH-dependent solubility. These properties make DOX an excellent model drug for validating the gatekeeping efficiency and pH-responsive release profile of chitosan-coated nanocarriers.

In recent years, nanotechnology and the development of nanocarriers have been considered in cancer therapy (Bhadran et al., 2025). Among various nanomaterials, mesoporous silica nanoparticles (MSNs) have attracted particular attention as ideal platforms for drug delivery due to their unique properties, such as excellent biocompatibility, very large internal surface area, high pore volume for loading large amounts of drugs, and ease of surface modification (Hou et al., 2026; Xu et al., 2023). However, one of the main challenges of using conventional silica nanoparticles is the risk of premature drug release into the bloodstream before reaching the target tissue (Nicolae et al., 2024; Vallet-Regí et al., 2018).

To address this challenge, stimuli-responsive drug delivery systems have been developed. Among the various types of stimuli, the design of pH-responsive systems is regarded as one of the most logical and efficient approaches in cancer therapy (AlSawaftah et al., 2022; Yilmaz et al., 2026). Due to the Warburg effect and high lactic acid production, the tumor microenvironment is mildly acidic (pH about 6.5–6.8). In addition, endosomes and lysosomes inside cancer cells have much higher acidity (pH about 5.0–5.5), while the bloodstream and healthy tissues have a physiological and neutral pH (7.4) (Pérez-Tomás and Pérez-Guillén, 2020; Worsley et al., 2022). This significant difference in acidity provides an excellent opportunity to design smart nanocarriers whose pores are blocked by coatings or molecular gatekeepers. These nanoparticles remain completely stable in the blood environment; however, upon entering the tumor microenvironment or after endocytosis by cancer cells, they undergo structural changes in response to the acidic conditions and release the encapsulated drug rapidly and precisely at the target site (Sun et al., 2023; Yang et al., 2015).

Generally, pH-responsive drug release from mesoporous silica nanoparticles can be achieved through two primary strategies. The first approach relies on the covalent conjugation of the drug to the nanoparticle surface via pH-sensitive cleavable bonds (e.g., hydrazone, acetal, or imine linkages). These bonds remain stable at physiological pH but rapidly hydrolyze in the acidic tumor microenvironment, releasing the free drug (Nigro et al., 2020). The second, and more widely adopted strategy, involves the physical blocking or capping of the mesopores using pH-responsive coatings or gatekeepers. In this strategy, the pores are sealed by polymers or supramolecular assemblies that undergo structural changes-such as swelling, dissolution, or electrostatic repulsion-upon protonation of functional groups (e.g., amino groups) in acidic conditions (Pasqua et al., 2013). In the present study, the latter strategy was adopted, employing a chitosan coating whose protonatable amino groups act as a pH-sensitive gatekeeper to seal the silica mesopores under physiological conditions and trigger drug release in the acidic tumor environment.

Recent studies have demonstrated the effectiveness of mesoporous silica–chitosan composite systems in drug delivery applications. Chitosan coating improves biocompatibility and acts as a pH-responsive gatekeeper, enhancing drug retention under physiological conditions and promoting release in acidic environments. Several studies have reported improved drug loading efficiency and controlled release behavior using such hybrid systems, highlighting their potential in targeted cancer therapy (Liu et al., 2023; Wang et al., 2023).

Given the high potential of nanomedicine to overcome current therapeutic limitations, the aim of this study was to design, synthesize, and evaluate a pH-sensitive mesoporous silica nanoparticle system coated with chitosan for targeted delivery of doxorubicin. Compared to previously reported chitosan-modified MSNs, the present system demonstrates a significant contrast in pH-responsive drug release (18% at pH 7.4 versus >76% at pH 5.5), which effectively minimizes premature drug leakage and enhances tumor-specific delivery. In addition, this study specifically evaluates therapeutic efficacy in head and neck squamous cell carcinoma (HNSCC), a relatively underexplored application for such nanocarriers. Furthermore, the synthesis approach is simple, reproducible, and based on biocompatible materials without the need for complex functionalization. The physicochemical properties, drug release behavior, and cytotoxic effects of the developed nanocarrier were systematically investigated to assess its potential as an effective platform for targeted chemotherapy. These aspects distinguish the present study from previously reported chitosan-based MSN systems.

## 2 Materials and methods

### 2.1 Materials

Tetraethyl orthosilicate (TEOS), cetyltrimethylammonium bromide (CTAB), sodium hydroxide (NaOH), chitosan (medium molecular weight, ∼50–190 kDa, degree of deacetylation ∼75–85%), acetic acid, and doxorubicin hydrochloride (DOX) were purchased from Sigma-Aldrich (St. Louis, MO, United States). DMEM culture medium, fetal bovine serum (FBS), penicillin/streptomycin, and trypsin were obtained from Gibco (Grand Island, NY, United States). Analytical grade salts for buffers and solvents were purchased from Merck (Darmstadt, Germany). Human head and neck squamous cell carcinoma (HNSCC) cell lines were obtained from the Pasteur Institute of Iran.

### 2.2 Synthesis of mesoporous silica nanoparticles (MSNs)

The sol–gel method was used to synthesize mesoporous silica nanoparticles with a surfactant template (Han et al., 2024). First, 1 g of CTAB was dissolved in 480 mL of deionized water. A specified amount of NaOH solution was then added to make the medium alkaline. The mixture was stirred continuously at 80 °C. After the temperature stabilized, 5 mL of TEOS was added dropwise to the solution, and the reaction was continued for 2 h. The resulting white precipitate was centrifuged and washed several times with deionized water and ethanol. To remove the CTAB template and open the pores, the nanoparticles were refluxed in a mixture of ethanol and hydrochloric acid (HCl) for 24 h. Finally, the nanoparticles (MSNs) were centrifuged, washed, and dried in a vacuum oven.

### 2.3 Doxorubicin loading and ph-sensitive coating (preparation of MSN-DOX@CS)

For drug loading, 50 mg of MSNs was dispersed in an aqueous doxorubicin solution, and the suspension was stirred for 24 h in the dark at room temperature to allow drug loading into the mesopores. Chitosan solution (dissolved in 1% acetic acid) was then added to the suspension and stirred for 12 h to allow chitosan to coat the nanoparticles and block the pores through electrostatic interactions. The final nanoparticles (MSN-DOX@CS) were centrifuged and washed with water. The supernatant was collected to determine the entrapment efficiency using a UV-Vis spectrophotometer (UV-1800, Shimadzu, Kyoto, Japan) at a wavelength of 480 nm.

### 2.4 Characterization of nanoparticles

To confirm the successful synthesis of nanoparticles, their morphology and size were examined using field emission scanning electron microscopy (FESEM,FEI, Hillsboro, OR, United States). The hydrodynamic size distribution and surface charge (zeta potential) of the particles before and after coating were measured using dynamic light scattering (DLS, Malvern Zetasizer Nano ZS90, United Kingdom). The chemical functional groups and the presence of chitosan and drug were confirmed using infrared spectroscopy (FTIR, Tensor II, Bruker, United States). To confirm successful drug loading and investigate potential molecular interactions between doxorubicin and the nanoparticle matrix, UV-Vis absorption spectra of free DOX (dissolved in deionized water at 10 μg/mL), empty MSN@CS nanoparticles (dispersed in deionized water at 100 μg/mL), and MSN-DOX@CS (dispersed in deionized water at an equivalent DOX concentration of 10 μg/mL) were recorded using a UV-Vis spectrophotometer (UV-1800, Shimadzu, Kyoto, Japan) over the wavelength range of 200–650 nm at a scan speed of 2 nm/s with a 1 cm quartz cuvette. Deionized water was used as the blank reference. All measurements were performed at room temperature.

### 2.5 Drug loading and *in vitro* drug release evaluation

The pH-responsive release behavior of MSN-DOX@CS was investigated using the dialysis bag diffusion method under sink conditions. To comprehensively evaluate the release profile across the pathophysiological pH range, four release media were prepared: phosphate-buffered saline (PBS, pH 7.4) to simulate blood circulation and healthy tissues; acetate buffers at pH 6.5 and pH 6.0 to mimic the mildly acidic extracellular tumor microenvironment; and acetate buffer at pH 5.5 to represent the acidic intracellular endo-lysosomal compartments. A defined amount of MSN-DOX@CS was dispersed in each buffer and sealed within a dialysis membrane (MWCO: 12–14 kDa). The bags were immersed in 50 mL of the corresponding release medium and incubated at 37 °C under continuous agitation (100 rpm). At predetermined time intervals, 3 mL aliquots were withdrawn and immediately replaced with an equal volume of fresh pre-warmed buffer to maintain sink conditions.

The concentration of released DOX in each aliquot was quantified by UV–Vis spectrophotometry (UV-1800, Shimadzu, Japan) at λ_max = 480 nm. Standard calibration curves were constructed in each buffer over the concentration range of 0.5–20 μg/mL, all exhibiting excellent linearity (R^2^ > 0.999) according to the Beer–Lambert law:

A=m·C+b

where A is the measured absorbance, C is the DOX concentration (µg/mL), m is the slope, and b is the y-intercept. The DOX concentration at each sampling time was calculated as:

$$C_{t}=\left(A_{t}-b\right)\;/\;m$$

The cumulative drug release percentage, corrected for aliquot replacement, was determined using:

$$\text{Cumulative Release }\left(\%\right)=\left[\left(V_{0}\times C_{t}\right)+\left(V_{s}\times \Sigma \;C_{i}\right)\right]/\;M_{\text{total}}\times 100$$

where V_0_ is the total release medium volume (50 mL), V_s_ is the sampling volume (3 mL), C_t_ is the DOX concentration at time t, Cᵢ is the DOX concentration at each previous sampling point (from i = 1 to t−1), and M_total is the total mass of DOX initially loaded into the nanoparticles (µg). Entrapment efficiency (EE) and loading capacity (LC) were calculated as:

$$\text{EE }\left(\%\right)=\left(W_{\text{initial}}-W_{\text{supernatant}}\right)\;/\;W_{\text{initial}}\times 100$$

$$\text{LC }\left(\%\right)=\left(W_{\text{initial}}-W_{\text{supernatant}}\right)\;/\;W_{\text{nanoparticles}}\times 100$$

where W_initial_ is the total DOX mass added during loading, W_supernatant_ is the unencapsulated DOX remaining in the supernatant, and W_nanoparticles_ is the total mass of drug-loaded nanoparticles.

### 2.6 Cytotoxicity assessment (MTT assay)

Human head and neck squamous cell carcinoma (HNSCC) cells were cultured in DMEM supplemented with 10% FBS and 1% penicillin/streptomycin and maintained at 37 °C in a humidified 5% CO_2_ atmosphere. To assess cell viability, cells were seeded at a density of 10,000 cells per well in 96-well plates and allowed to adhere for 24 h. The cells were then treated with various concentrations of free drug (Free DOX), drug-free nanoparticles (MSN@CS, as a negative control), and drug-loaded nanoparticles (MSN-DOX@CS) for 24 and 48 h. At the end of each treatment period, the culture medium was carefully removed and replaced with 100 µL of MTT solution (0.5 mg/mL), freshly prepared by diluting a 5 mg/mL MTT stock solution (dissolved in sterile PBS, pH 7.4, and filter-sterilized through a 0.22 µm membrane) at a 1:10 ratio with serum-free DMEM. The plates were incubated for 4 h at 37 °C in a humidified 5% CO_2_ atmosphere to allow the reduction of MTT to insoluble purple formazan crystals by the mitochondrial dehydrogenases of viable cells. Subsequently, the MTT solution was aspirated, and 150 µL of dimethyl sulfoxide (DMSO) was added to each well to solubilize the formazan crystals. After 10 min of gentle shaking at room temperature, the absorbance was measured at 570 nm (reference wavelength: 630 nm) using a microplate reader. Prior to the main experiments, the assay was optimized by evaluating different MTT concentrations (0.25, 0.5, and 1.0 mg/mL) and incubation times (2, 4, and 6 h); the combination of 0.5 mg/mL MTT and 4 h incubation yielded the highest signal-to-noise ratio and a linear correlation between cell number and absorbance, and was therefore adopted for all experiments. A blank control (medium + MTT without cells) was included to correct for background absorbance, and to rule out any direct interference of the nanoparticles with the MTT reagent, MSN@CS was added to cell-free wells at the highest test concentration, confirming no significant absorbance at 570 nm. Cell viability was calculated as:

$$\text{Cell Viability }\left(\%\right)=\left(A_{\text{sample}}-A_{\text{blank}}\right)\;/\;\left(A_{\text{control}}-A_{\text{blank}}\right)\times 100$$

where A_sample_ is the absorbance of treated cells, A_control_ is the absorbance of untreated cells, and A_blank_ is the absorbance of the cell-free control.

To evaluate the biocompatibility and selective toxicity of the formulation, the MTT assay was additionally performed on HaCaT (human keratinocyte) cells as a non-cancerous control. Cells were cultured under standard conditions and treated with equivalent concentrations of Free DOX, empty MSN@CS, and MSN-DOX@CS for 24 and 48 h, following the same protocol described above. All experiments were performed in triplicate and results are reported as mean ± SD.

### 2.7 Statistical analysis

All experiments were performed in at least three independent replicates, and results were reported as mean ± standard deviation (mean ± SD). GraphPad Prism software (version 9.0, GraphPad Software, San Diego, CA, United States) was used for statistical analysis. Comparisons between groups were performed using analysis of variance (ANOVA) and Tukey’s *post hoc* test. *p* values less than 0.05 (*p* < 0.05) were considered statistically significant.

## 3 Results

### 3.1 Design and synthesis of the MSN-DOX@CS nanocarrier

The overall synthesis strategy and the proposed pH-responsive mechanism of the MSN-DOX@CS system are schematically illustrated in Figure 1. Initially, MSNs were synthesized via the sol–gel method, generating a porous framework with a high surface area. Subsequently, DOX was loaded into the mesopores through diffusion and electrostatic interactions between the positively charged amino groups of DOX and the negatively charged silanol (Si–OH) groups of the silica surface. Finally, the cationic chitosan polymer was electrostatically deposited onto the nanoparticle surface, sealing the pores and acting as a pH-sensitive molecular gatekeeper. As depicted in Figure 1, the release behavior of the system is governed by environmental pH. Under physiological conditions (pH 7.4), the chitosan coating remains compact and intact, effectively blocking the pores and preventing premature drug leakage into the bloodstream. However, upon reaching the acidic tumor microenvironment or following cellular internalization into acidic endosomes (pH 5.5), the amino groups of chitosan (–NH_2_) become protonated (–NH_3_^+^). This protonation induces strong electrostatic repulsion between the polymer chains, leading to swelling and partial dissolution of the chitosan layer. Consequently, the pores are opened, triggering the rapid and targeted release of doxorubicin at the tumor site.

**FIGURE 1:**
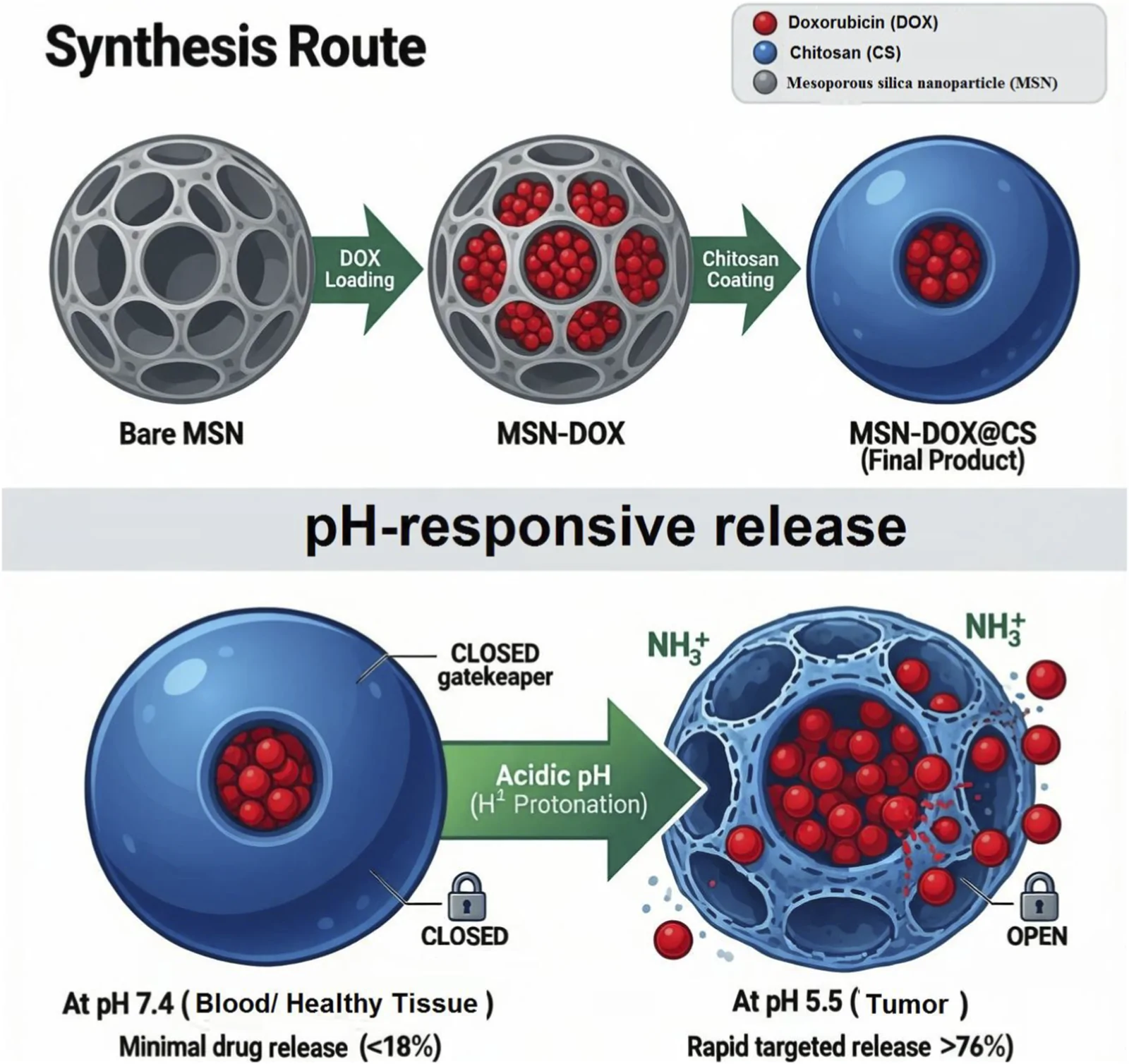
Schematic representation of the synthesis route and pH-responsive drug release mechanism of the MSN-DOX@CS nanocarrier. The synthesis involves three sequential steps (Barsouk et al., 2023): preparation of bare mesoporous silica nanoparticles (MSNs) (Mordzińska-Rak et al., 2025), loading of doxorubicin (DOX) into the mesopores, and (Ganesan et al., 2024) coating with chitosan (CS) to seal the pores. The lower panel illustrates the release mechanism: at physiological pH (7.4), the intact chitosan layer keeps the “gatekeeper” closed, resulting in minimal drug release (∼18%). In contrast, at acidic pH (5.5), the protonation of chitosan amino groups (–NH_3_^+^) causes electrostatic repulsion and polymer swelling, opening the gatekeeper and enabling rapid drug release (>76%).

### 3.2 Physicochemical characterization of nanoparticles

To investigate the morphology and size of the synthesized nanoparticles, FESEM electron microscopes were used. As shown in Figure 2, the mesoporous silica nanoparticles (Bare MSNs) exhibit a uniform semi-spherical shape with an appropriate size distribution (diameter of about 100–150 nm). After drug loading and coating with chitosan (MSN-DOX@CS), the spherical morphology was retained; however, due to the formation of a polymer layer, the particle edges became slightly less defined and their surface roughness increased.

**FIGURE 2:**
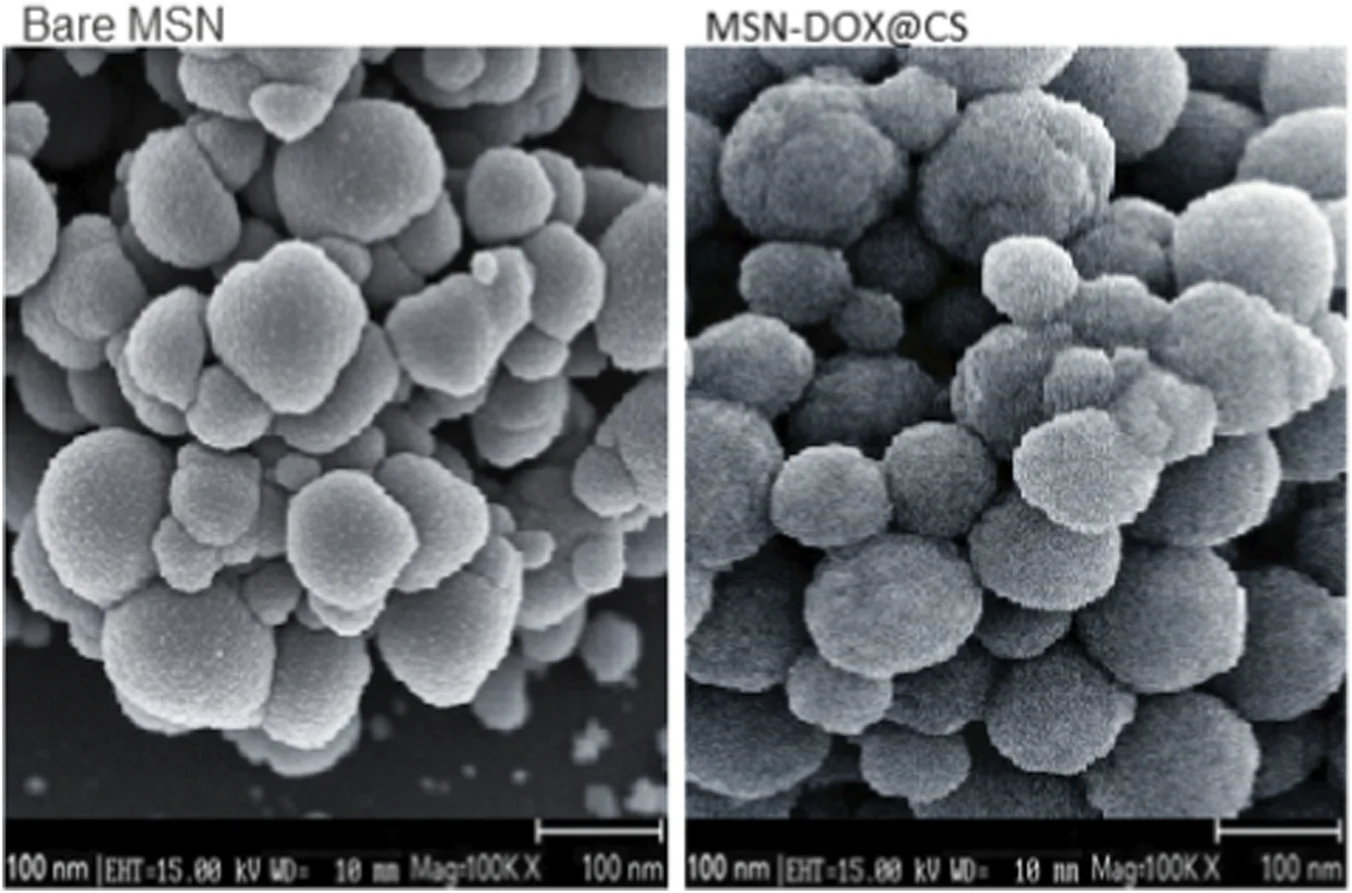
FESEM image of MSN and MSN-DOX@CS nanoparticles.

To confirm the success of the surface modification steps, changes in surface charge (zeta potential) and hydrodynamic size (DLS) were evaluated (Figure 3; Table 1). Uncoated silica nanoparticles (MSNs) exhibited a strongly negative surface charge (−25.4 mV) due to the presence of silanol (Si-OH) groups. After coating with the cationic polymer chitosan, the zeta potential changed significantly, reaching a positive value (+28.6 mV), confirming the successful deposition of chitosan on the nanoparticle surface. Additionally, the average hydrodynamic size of the particles increased from 112 nm for MSN to 145 nm for MSN-DOX@CS.

**FIGURE 3:**
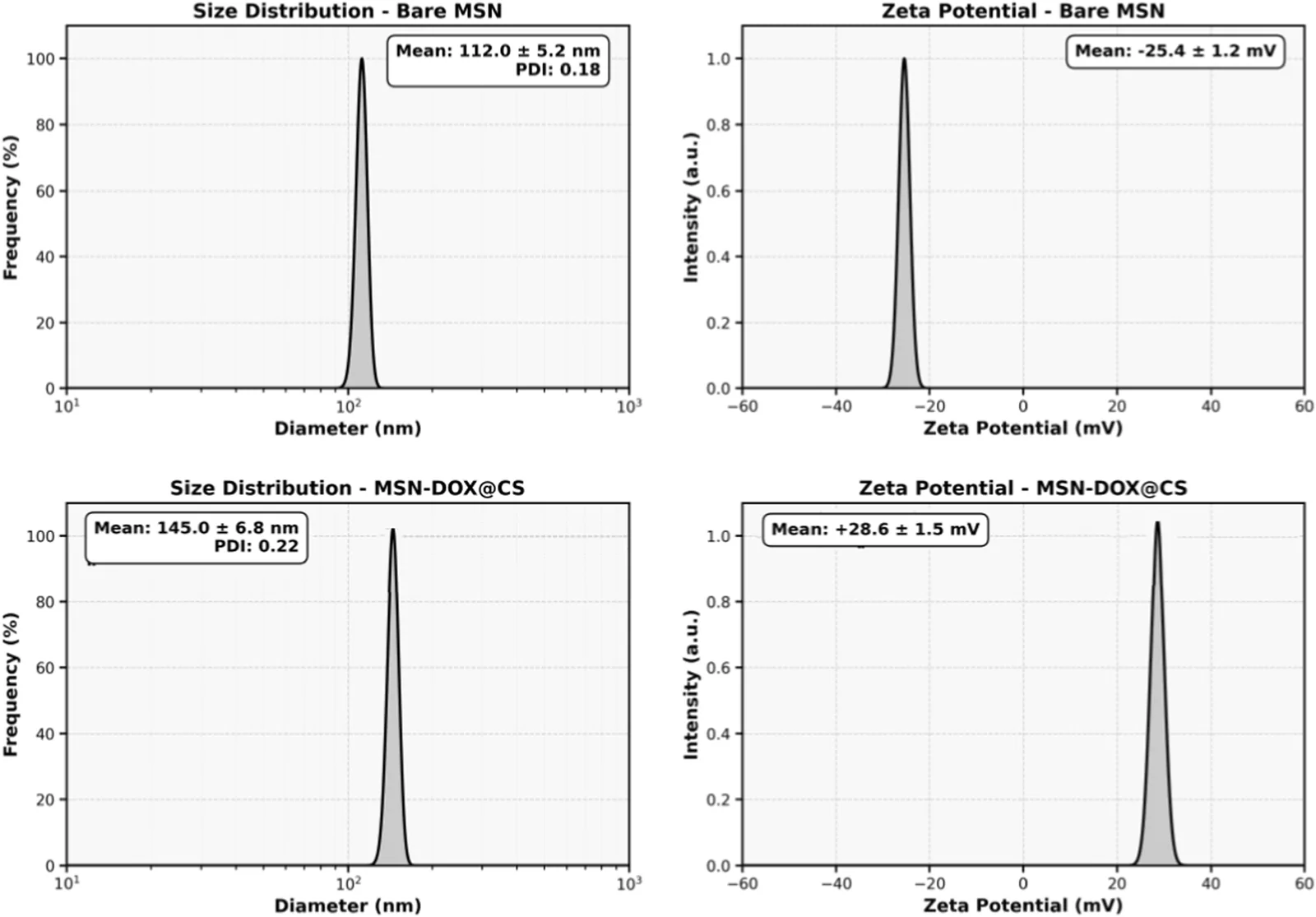
Size characteristics and surface charge (Zeta Potential) of nanoparticles.

**TABLE 1:** Size characteristics and surface charge (Zeta Potential) of nanoparticles.

Sample	Hydrodynamic size (nm)	PDI	Zeta potential (mV)
Bare MSN	112 ± 5.2	0.18	−25.4 ± 1.2
MSN-DOX@CS	145 ± 6.8	0.22	+28.6 ± 1.5

FTIR spectroscopy was employed to confirm the chemical composition of each individual component and the final composite nanocarrier (Figure 4). The spectrum of bare MSNs exhibited characteristic silica bands, including a broad absorption at ∼3,430 cm^−1^ attributed to O–H stretching vibrations of surface silanol groups and adsorbed water molecules, a strong band at ∼1,080 cm^−1^ corresponding to Si–O–Si asymmetric stretching, a band at ∼800 cm^−1^ assigned to Si–O–Si symmetric stretching, and a band at ∼460 cm^−1^ related to O–Si–O bending vibrations. The spectrum of free DOX displayed several diagnostic peaks associated with its anthracycline tetracyclic ring system and functional groups. The broad band at ∼3,430 cm^−1^ corresponds to O–H and N–H stretching vibrations of the hydroxyl and primary amine groups, while C–H stretching vibrations of the aliphatic moieties appear at ∼2,925 cm^−1^. The carbonyl stretching vibration of the ketone group on the acetyl side chain (C-13) is observed at ∼1730 cm^−1^, and the quinone C=O stretching of the anthracycline ring system (C-5 and C-12) appears at ∼1,618 cm^−1^. The aromatic C=C stretching vibrations of the tetracyclic anthracycline core are evident at ∼1,580 cm^−1^, and the N–H bending vibration of the primary amine group on the daunosamine sugar moiety is observed at ∼1,525 cm^−1^. Additional bands at ∼1,283 cm^−1^ and ∼990 cm^−1^ correspond to C–O stretching and C–O–C glycosidic linkage vibrations of the daunosamine sugar, respectively. The spectrum of pure chitosan showed a broad band at ∼3,430 cm^−1^ (O–H and N–H stretching), C–H stretching vibrations at ∼2,920 cm^−1^, the characteristic Amide I band (C=O stretching) at ∼1,655 cm^−1^, the Amide II band (N–H bending) at ∼1,595 cm^−1^, C–O–C glycosidic bond stretching at ∼1,155 cm^−1^, and C–O skeletal vibrations at ∼1,075 cm^−1^. In the spectrum of the final MSN-DOX@CS composite, all characteristic bands of the individual components are clearly present, confirming successful drug loading and polymer coating. The silica backbone is confirmed by the Si–O–Si bands at ∼1,080 and ∼800 cm^−1^. The chitosan coating is evidenced by the Amide I band at ∼1,655 cm^−1^ and C–H stretching at ∼2,920 cm^−1^. Importantly, the anthracycline ring signatures of doxorubicin are distinctly visible in the composite spectrum: the C=O ketone band at ∼1730 cm^−1^ and the aromatic C=C band at ∼1,580 cm^−1^, which are absent in both the bare MSN and pure chitosan spectra, unequivocally confirming the successful encapsulation of DOX within the mesoporous silica framework and the effective deposition of the chitosan gatekeeping layer on the nanoparticle surface.

**FIGURE 4:**
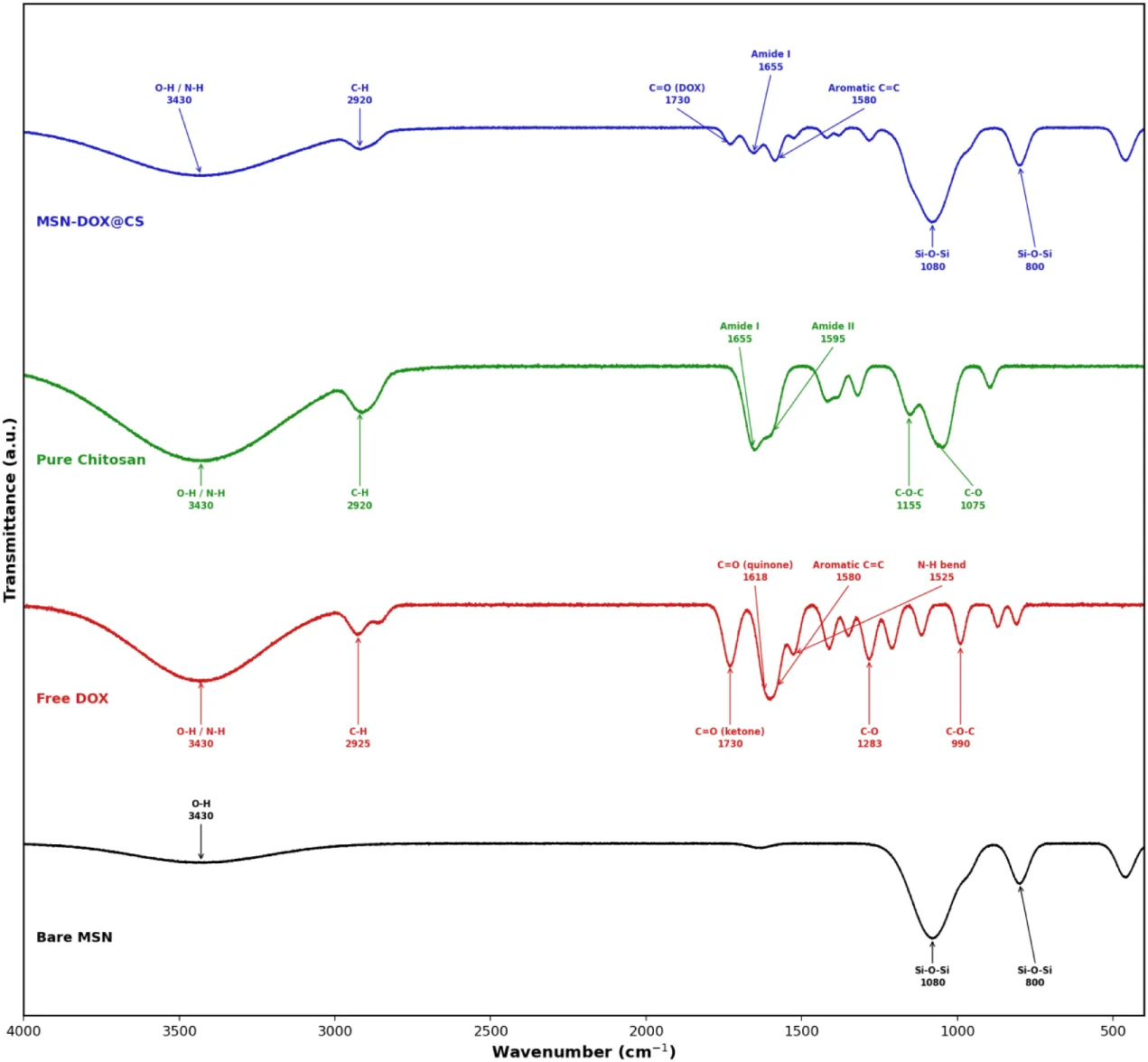
FTIR spectra of bare mesoporous silica nanoparticles (Bare MSN, black), free doxorubicin (Free DOX, red), pure chitosan (Pure Chitosan, green), and the final chitosan-coated doxorubicin-loaded nanoparticles (MSN-DOX@CS, blue). The bare MSN spectrum shows characteristic Si–O–Si stretching bands at 1,080 and 800 cm^−1^ and O–H stretching at 3,430 cm^−1^. The free DOX spectrum exhibits diagnostic anthracycline ring peaks, including C=O ketone (1730 cm^−1^), C=O quinone (1,618 cm^−1^), aromatic C=C (1,580 cm^−1^), and N–H bending (1,525 cm^−1^). The pure chitosan spectrum displays Amide I (1,655 cm^−1^) and Amide II (1,595 cm^−1^) bands. The MSN-DOX@CS composite spectrum contains all characteristic peaks of the individual components, confirming successful drug loading and chitosan coating.

### 3.3 Drug loading evaluation

Using UV-Vis analysis, the entrapment efficiency (EE) of doxorubicin was calculated as 78.5%, and the loading capacity (LC) was calculated as 15.2%. UV-Vis absorption spectra of free DOX, empty MSN@CS, and MSN-DOX@CS were recorded to confirm drug loading and investigate drug–matrix interactions (Figure 5). Free DOX exhibited characteristic absorption bands at 232 and 252 nm (π→π* transitions of the anthracycline aromatic rings), 290 nm (n→π* transition of quinone moieties), and 480 and 530 nm (n→π* transition of the quinone chromophore). The empty MSN@CS showed negligible absorption, confirming the absence of interfering chromophores. In contrast, MSN-DOX@CS displayed all DOX absorption bands with a slight red shift of ∼3 nm (232 → 235 nm and 480 → 483 nm) and moderate peak broadening, attributed to interactions between the amino/hydroxyl groups of DOX and the silanol groups of silica as well as the functional groups of chitosan. The elevated baseline below 350 nm is consistent with nanoparticle light scattering. These results confirm successful DOX encapsulation and drug–matrix interactions that may contribute to the pH-responsive release behavior.

**FIGURE 5:**
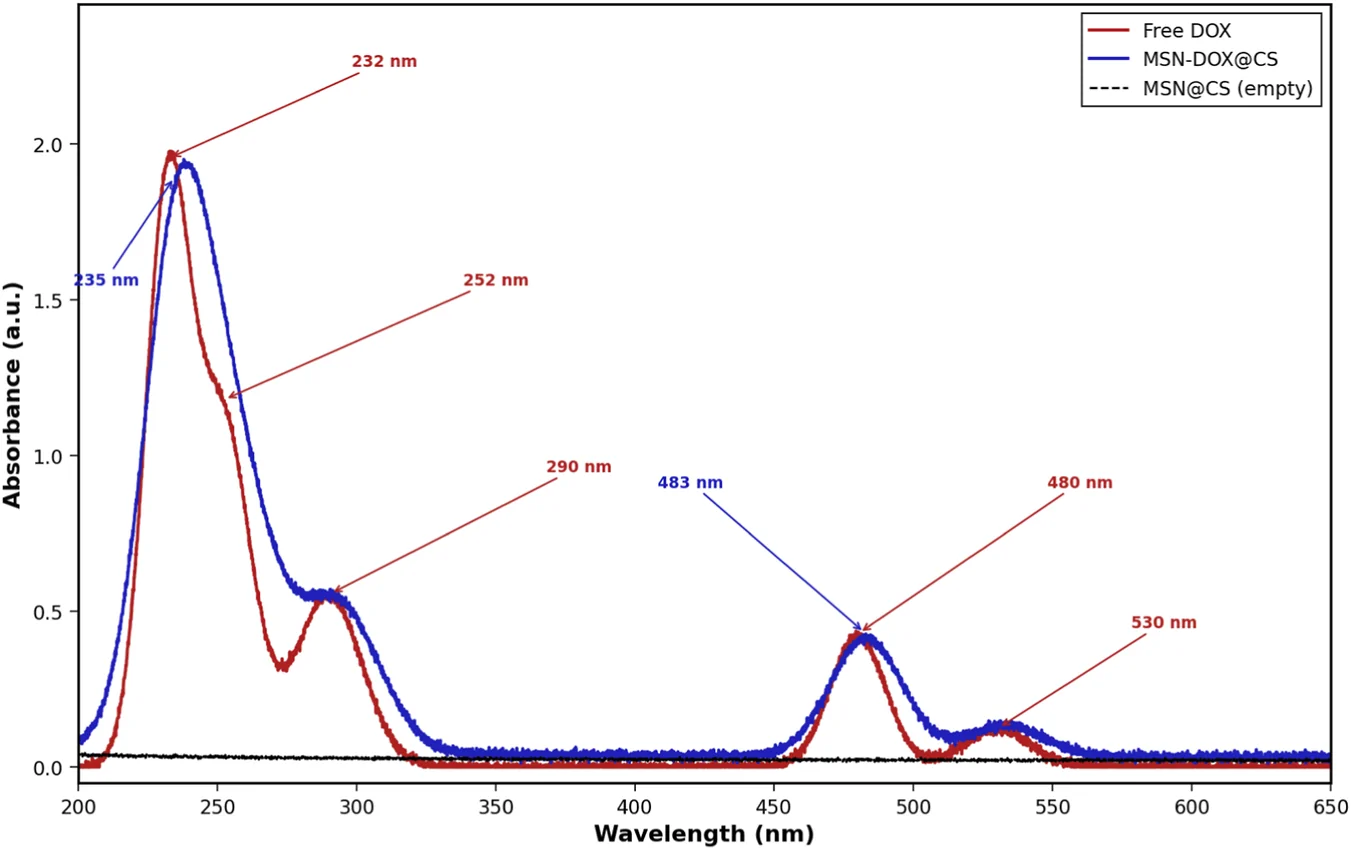
UV-Vis absorption spectra of free doxorubicin (Free DOX, red solid line), the drug-loaded nanocarrier (MSN-DOX@CS, blue solid line), and the empty nanocarrier (MSN@CS, black dashed line) recorded over the wavelength range of 200–650 nm. The free DOX spectrum displays characteristic anthracycline absorption bands at 232, 252, and 290 nm (UV region) and at 480 and 530 nm (visible region). The MSN-DOX@CS composite spectrum exhibits the same absorption features with a slight red shift (∼3 nm; 232 → 235 nm and 480 → 483 nm) and peak broadening, confirming successful drug loading and molecular interactions between DOX and the silica–chitosan matrix. The empty MSN@CS carrier shows negligible absorption across the entire range, demonstrating the absence of interfering chromophores.

### 3.4 pH-responsive release profile

To simulate the entire pathophysiological pH gradient-ranging from blood circulation to the intracellular compartments-drug release was evaluated at four distinct pH values (7.4, 6.5, 6.0, and 5.5). As shown in Figure 6, the system exhibited a clear pH-dependent release behavior. While minimal release occurred at physiological pH 7.4 (18%), the release rate increased incrementally in the mildly acidic tumor extracellular conditions (36% at pH 6.5% and 51% at pH 6.0), reaching a maximum burst release (>76%) at the endosomal pH of 5.5. This progressive response confirms the sensitivity of the chitosan gatekeeper to the subtle pH variations present in the tumor microenvironment.

**FIGURE 6:**
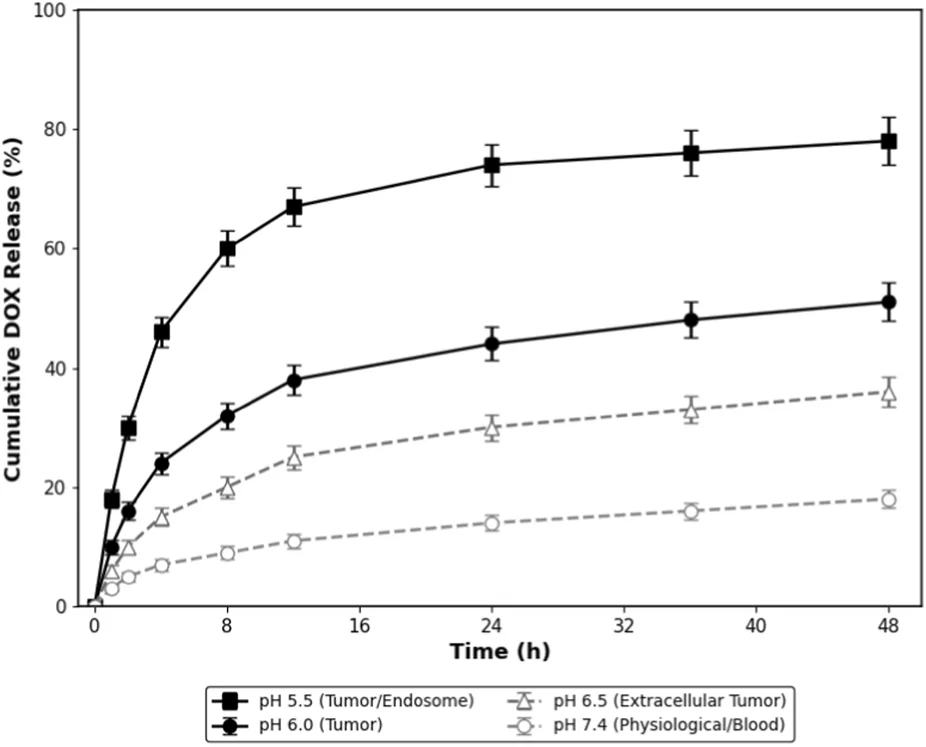
Cumulative release profile of doxorubicin from MSN-DOX@CS nanoparticles in two environments with physiological pH (7.4) and acidic tumor pH (5.5) over a period of 48 h.

### 3.5 *In vitro* cytotoxicity assessment

The therapeutic effect of nanoparticles on HNSCC cell viability was assessed using the MTT assay (Figure 7). The results showed that even at high concentrations (100 μg/mL), more than 90% of the cells survived, demonstrating the excellent biocompatibility and intrinsic non-toxicity of the silica nanoparticles and chitosan coating. Cells were then treated with equivalent concentrations of free drug (Free DOX) and drug-loaded nanoparticles (MSN-DOX@CS). The results indicated that both groups reduced cancer cell viability in a dose-dependent manner. At low concentrations, the free drug acted slightly faster, likely due to passive diffusion, but at higher concentrations and at 48 h, MSN-DOX@CS nanoparticles exhibited very high cytotoxicity, resulting in a statistically significant difference (*p* < 0.05). This high efficacy is attributed to efficient cellular uptake via endocytosis, high intracellular drug accumulation, and targeted, burst release of the drug in response to the acidic intracellular environment, which may help reduce the impact of drug efflux mechanisms in cancer cells.

**FIGURE 7:**
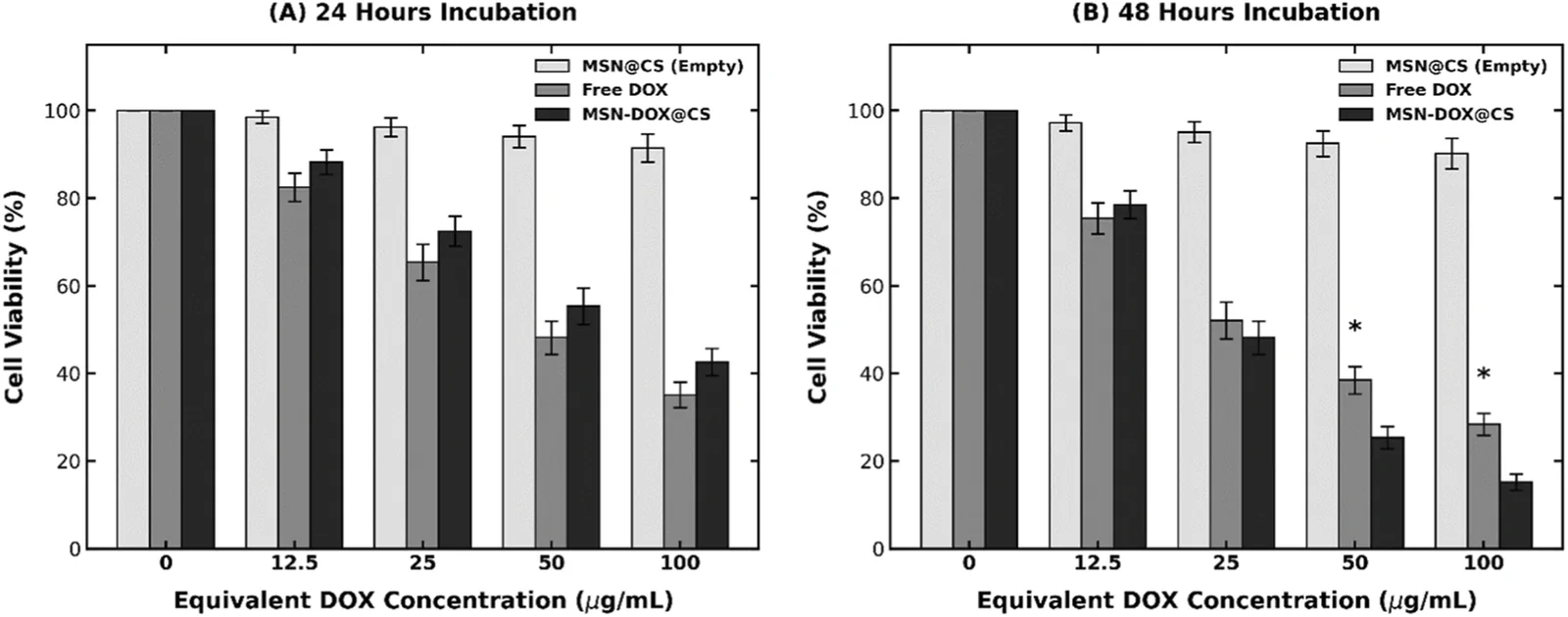
Percentage of HNSCC cell viability after 24 **(A)** and 48 h **(B)** of treatment with empty nanoparticles (negative control), free DOX, and MSN-DOX@CS nanoparticles at different DOX concentrations, based on MTT assay (*p < 0.05, statistically significant compared to the control group).

To confirm the safety profile of the developed nanocarrier, cytotoxicity was assessed on HaCat cell. The empty nanocarrier (MSN@CS) demonstrated biocompatibility, with cell viability exceeding 103% even at 100 μg/mL, indicating that the silica-chitosan matrix itself is non-toxic to healthy cells. In contrast, free DOX significantly reduced normal cell viability in a dose-dependent manner. Notably, MSN-DOX@CS exhibited significantly lower cytotoxicity toward normal cells compared to free DOX at equivalent concentrations (p < 0.05). This reduced toxicity is attributed to the chitosan gatekeeper, which remains stable at physiological pH and prevents premature drug release into healthy tissues (Figure 8).

**FIGURE 8:**
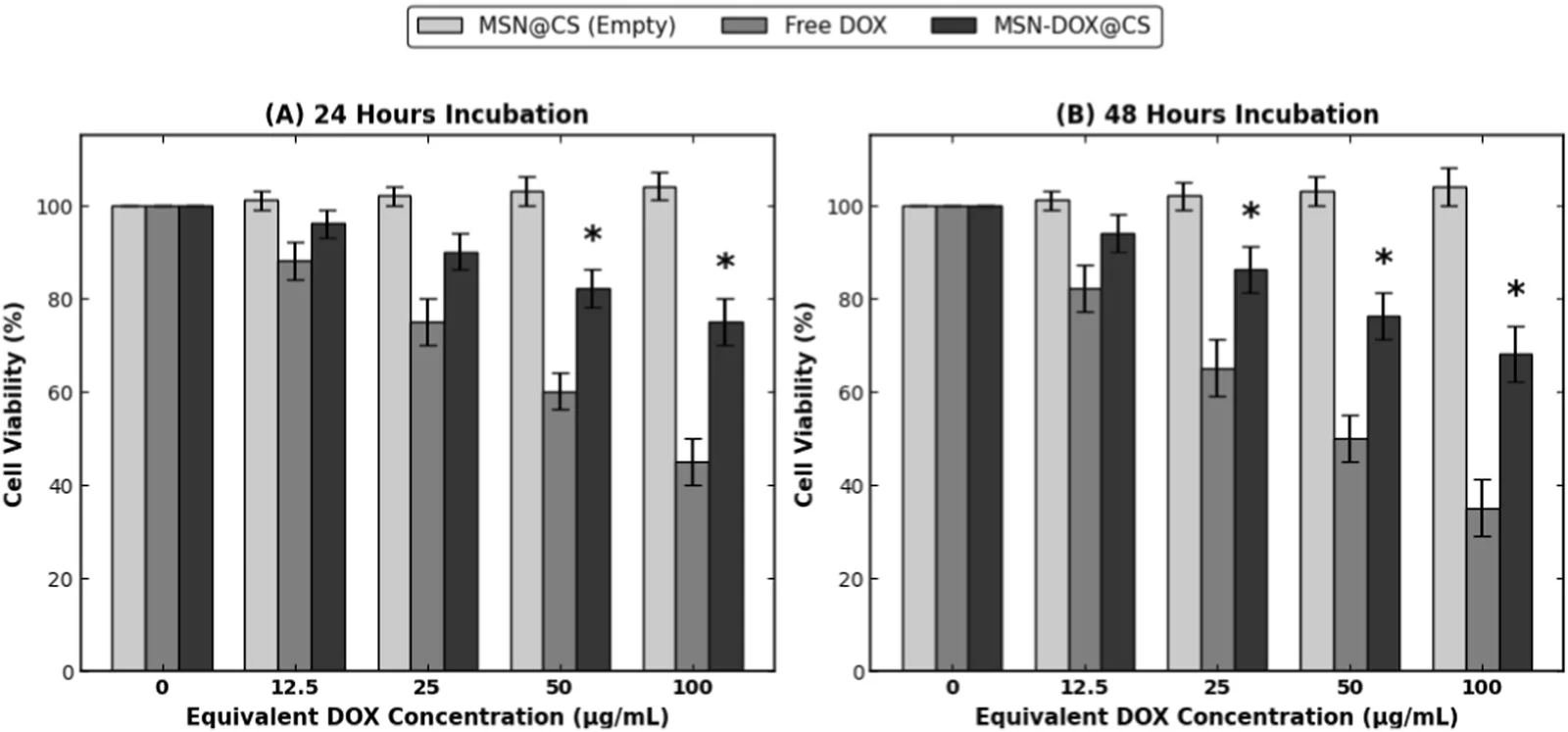
Cell viability of normal (HaCat) cells after **(A)** 24 h and **(B)** 48 h of treatment with empty nanoparticles (MSN@CS), free DOX, and MSN-DOX@CS at different concentrations. The empty carrier showed excellent biocompatibility (>100% viability). Notably, MSN-DOX@CS exhibited significantly reduced toxicity toward normal cells compared to Free DOX (p < 0.05), confirming the selective safety profile of the pH-responsive system.

## 4 Discussion

According to the results of this study, the designed nanocarrier, based on mesoporous silica nanoparticles coated with chitosan as a targeted drug delivery platform responsive to pH changes, demonstrates highly effective performance in the controlled release of doxorubicin in the acidic environment of tumors. The presence of chitosan plays a crucial role in the performance of the developed nanocarrier. Due to its cationic nature, chitosan interacts with the negatively charged silica surface and acts as a gatekeeping layer, preventing premature drug leakage at physiological pH. Under acidic conditions, protonation of chitosan leads to structural changes that facilitate drug release. This behavior highlights the importance of chitosan in achieving controlled and targeted drug delivery. While maintaining stability under physiological blood conditions (pH 7.4), it selectively and rapidly releases the drug in the acidic intracellular environment (pH 5.5). This property reduces systemic side effects and increases selective toxicity against head and neck cancer cells (HNSCC) (Shakeran et al., 2021; Gisbert-Garzarán et al., 2017). The results of the physicochemical characterization of the nanoparticles (FESEM, DLS analysis, and FTIR spectroscopy) confirmed the successful synthesis, drug loading, and surface coating with chitosan. The change in zeta potential from a strong negative value (−25.4 mV) in empty nanoparticles to a positive value (+28.6 mV) after chitosan coating indicates the formation of a stable electrostatic layer on the surface, which, in addition to effectively controlling release, can facilitate cellular uptake through interaction with the negatively charged membranes of cancer cells. These findings are consistent with those of similar studies (Kim et al., 2008; Marques Gonçalves et al., 2023; Myat et al., 2025). Furthermore, the increase in hydrodynamic size from 112 nm to 145 nm and the appearance of characteristic peaks of chitosan and doxorubicin in the FTIR spectrum confirm successful loading and coating. The most important finding of this study is the smart drug release behavior in two different pH environments. Only about 18% of the drug was released at neutral pH (blood simulation) after 48 h, indicating the effective function of chitosan in slowing drug release under physiological conditions. In contrast, at acidic pH (5.5), more than 76% of the drug was released, which is attributed to the protonation of the amino groups of chitosan, the creation of electrostatic repulsion, swelling and dissolution of the polymer layer, resulting in pore opening and increased drug solubility in an acidic environment. In addition to the protonation of chitosan amino groups, other physicochemical factors may contribute to the observed pH-responsive behavior. Under acidic conditions, doxorubicin becomes more protonated, which increases its solubility and facilitates its diffusion from the nanoparticle pores. Furthermore, chitosan undergoes swelling and partial dissolution at lower pH, promoting pore opening and accelerating drug release. The mesoporous structure of silica nanoparticles also contributes to diffusion-controlled release behavior, which becomes more pronounced as the polymer barrier weakens under acidic conditions. This mechanism has also been investigated and confirmed in similar studies (Shikuku et al., 2024; Rizwan et al., 2017; Du et al., 2015; Stefanache et al., 2025). In the evaluation of cytotoxicity, the empty nanocarrier (MSN@CS) showed more than 90% cell survival even at high concentrations, indicating the excellent biocompatibility of silica and chitosan, which is vital for clinical applications. In the comparison between the free drug and the drug nanocarrier, although the free drug was slightly more effective in the first 24 h due to direct penetration and passive diffusion, at 48 h, MSN-DOX@CS showed statistically significant superiority (*p* < 0.05) in reducing cell survival, indicating greater intracellular accumulation of the drug through endocytosis and targeted release in the endosome/lysosome, which may reduce the impact of drug resistance mechanisms and contribute to improved therapeutic efficacy (Ahmadi et al., 2022; Yap et al., 2021). In this study, the nanocarrier was synthesized from biocompatible, low-cost materials without the use of complex ligands, so that the designed nanocarrier achieved a clear distinction in release between blood and tumor environments. The selection of materials-silica and chitosan-is driven by their well-established biocompatibility profile. Mesoporous silica nanoparticles have demonstrated favorable safety profiles in preclinical models, characterized by low immunogenicity and efficient renal clearance (Vallet-Regí et al., 2007). Similarly, chitosan, a natural polysaccharide derived from chitin, is widely employed in biomedical applications due to its non-toxicity, biodegradability, and excellent mucosal tolerance (Kim et al., 2023). Furthermore, unlike conventional targeted delivery systems that often rely on complex, expensive, and potentially unstable covalent conjugation of ligands (e.g., monoclonal antibodies or aptamers), our nanocarrier used a simple and robust electrostatic assembly (Jiang et al., 2015; Alsarayreh et al., 2026). This ligand-free approach significantly reduces production costs, minimizes the risk of structural degradation during synthesis, and circumvents the potential immunogenicity associated with synthetic targeting moieties. By combining these low-cost, readily available materials with a straightforward sol-gel synthesis and simple physical coating method, our platform achieves a high-performance, pH-responsive drug delivery system that is both scalable and suitable for clinical development.

The selectivity of an anticancer nanocarrier is demonstrated not only by its potency against malignant cells but also by its safety towards healthy tissues. In this study, the evaluation of MSN-DOX@CS in a normal cell line revealed that the empty nanocarrier is inherently biocompatible, while the drug-loaded formulation exerts significantly lower toxicity in non-cancerous cells compared with free DOX. This selectivity arises directly from the pH-responsive design of the chitosan coating: under the neutral pH of healthy tissues and the bloodstream, the polymer gatekeeper remains intact, minimizing premature drug leakage and systemic exposure. Conversely, upon internalization into cancer cells and exposure to acidic endosomal/lysosomal conditions, the gatekeeper opens to facilitate rapid, targeted drug release. The favorable safety profile in normal cells, combined with the enhanced cytotoxicity observed in HNSCC cells, underscores the clinical translational potential of the MSN-DOX@CS platform for improving the therapeutic index of doxorubicin.

The selected doxorubicin loading conditions were optimized to achieve sufficient drug encapsulation while maintaining nanoparticle stability and controlled release properties. The relatively high loading efficiency obtained in this study demonstrates the capability of mesoporous silica nanoparticles to effectively accommodate therapeutic agents without compromising system performance.

One limitation of the present study is the lack of direct cellular internalization and trafficking analysis, such as confocal microscopy, which could provide more detailed insight into nanoparticle uptake and intracellular drug localization. Future studies will focus on these aspects to further elucidate the mechanism of action.

## 5 Conclusion

Overall, this study demonstrated that the MSN-DOX@CS system can serve as a smart and effective nanocarrier for targeted treatment of head and neck cancer. By combining the structural advantages of MSN with the pH-dependent responsiveness of chitosan, it not only addresses the problem of early drug leakage, but also improves therapeutic efficacy and reduces systemic side effects by increasing intracellular accumulation and enabling controlled release.

## Data Availability

The original contributions presented in the study are included in the article/supplementary material, further inquiries can be directed to the corresponding authors.
